# A novel close-circulating vapor stripping-vapor permeation technique for boosting biobutanol production and recovery

**DOI:** 10.1186/s13068-018-1129-5

**Published:** 2018-05-04

**Authors:** Chao Zhu, Lijie Chen, Chuang Xue, Fengwu Bai

**Affiliations:** School of Life Science and Biotechnology, Dalian University of Technology, No 2 Linggong Road, Dalian, 116024 China

**Keywords:** Butanol, Vapor stripping-vapor permeation, ABE fermentation, In situ product recovery

## Abstract

**Background:**

Butanol derived from renewable resources by microbial fermentation is considered as one of not only valuable platform chemicals but alternative advanced biofuels. However, due to low butanol concentration in fermentation broth, butanol production is restricted by high energy consumption for product recovery. For in situ butanol recovery techniques, such as gas stripping and pervaporation, the common problem is their low efficiency in harvesting and concentrating butanol. Therefore, there is a necessity to develop an advanced butanol recovery technique for cost-effective biobutanol production.

**Results:**

A close-circulating vapor stripping-vapor permeation (VSVP) process was developed with temperature-difference control for single-stage butanol recovery. In the best scenario, the highest butanol separation factor of 142.7 reported to date could be achieved with commonly used polydimethylsiloxane membrane, when temperatures of feed solution and membrane surroundings were 70 and 0 °C, respectively. Additionally, more ABE (31.2 vs. 17.7 g/L) were produced in the integrated VSVP process, with a higher butanol yield (0.21 vs. 0.17 g/g) due to the mitigation of butanol inhibition. The integrated VSVP process generated a highly concentrated permeate containing 212.7 g/L butanol (339.3 g/L ABE), with the reduced energy consumption of 19.6 kJ/g-butanol.

**Conclusions:**

Therefore, the present study demonstrated a well-designed energy-efficient technique named by vapor stripping-vapor permeation for single-stage butanol removal. The butanol separation factor was multiplied by the temperature-difference control strategy which could double butanol recovery performance. This advanced VSVP process can completely eliminate membrane fouling risk for fermentative butanol separation, which is superior to other techniques.

**Electronic supplementary material:**

The online version of this article (10.1186/s13068-018-1129-5) contains supplementary material, which is available to authorized users.

## Background

With the inevitable depletion of fossil fuels and increase of environmental issues, it’s essential to develop renewable and clean energy sources [1, 2]. As a substitute for petroleum fuel, biobutanol derived from renewable resources, is superior to bioethanol because of its favorable physico-chemical and fuel properties [3, 4]. Currently, the butanol produced by ABE fermentation is preferentially applied in food and pharmaceutical industry [5], but as fuel substitute it still lacks economic competitiveness due to high energy consumption for product recovery induced by the low butanol titer in fermentation broth [6]. Thus, increasing attentions have been paid to the development of butanol recovery processes for addressing energy consumption issue [2, 7].

The techniques for in situ butanol recovery from fermentation system to alleviate butanol inhibition and improve butanol productivity can be classified as: (i) based on introduction of additional materials: adsorption (adsorbents) [8, 9], liquid–liquid extraction (reagents) [10, 11], and gas stripping (GS) (carrier gas) [12, 13]; and (ii) based on usage of permselective membrane: pervaporation (PV) (permeative membrane) [14–16]. But the common problem of above-mentioned methods is their low efficiency in harvesting and concentrating butanol. GS has the significant advantages of simple scale up, easy operation, only removal of volatile compounds etc., but the ABE (butanol) titers recovered via GS in previous studies were less than 230 g/L (180 g/L butanol), resulting in high energy requirement during current product recovery and subsequent purification [4, 17]. PV, based on the rapidly developing membrane technology, has great potentials in butanol recovery integrated with ABE fermentation, but its performance is seriously governed by the structure and properties of membrane. Moreover, the contact between membrane surface and ABE fermentation broth will inevitably lead to membrane contamination induced by the adsorption of cells, ions, sugars, biomacromolecules and so on, which usually leads to the decreases of flux and selectivity in a long-term operation and extra treatment such as membrane cleaning [18, 19]. Therefore, there is an urgent need for developing an advanced process for butanol recovery superior to these above-mentioned techniques.

**Fig. 1:**
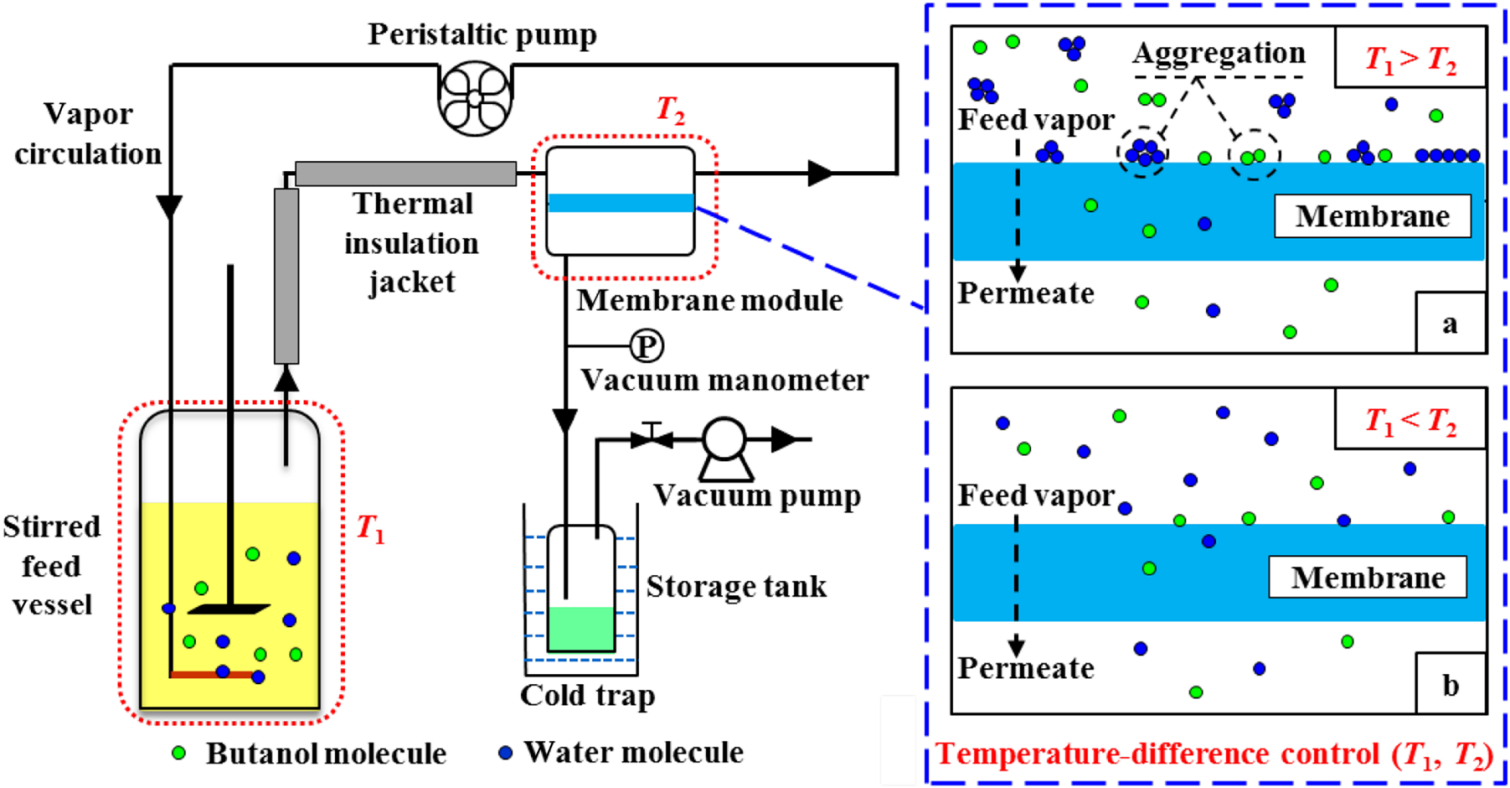
Experimental apparatus for butanol recovery with close-circulating VSVP process with temperature-difference control. **a** The status of feed vapor molecules in membrane module at *T*_1_ > *T*_2_. **b** The status of feed vapor molecules in membrane module at *T*_1_ < *T*_2_

The polydimethylsiloxane (PDMS) is widely used in PV process, which be considered a benchmark for its good thermal and mechanical stability with excellent performance [14, 20]. To develop an efficient technique for butanol recovery, a novel single-stage vapor stripping-vapor permeation (VSVP) process with close-circulating stripping gas was systematically characterized using homogenous PDMS membrane as shown in Fig. 1. The effects of membrane thickness, gas flow rate, and temperature difference between feed solution and membrane surroundings were investigated for improvement of separation performance. Furthermore, the close-circulating VSVP process integrated with ABE fermentation and its energy requirement were also evaluated and compared with other studies.

## Results and discussion

### Effects of membrane thickness and gas flow rate on VSVP performance

For the membrane-based separation process, the path of permeation for feed components is determined by the thickness of a dense membrane. The effect of membrane thickness on the performance of VSVP process is shown in Table 1. The feed vessel loaded with butanol binary solution as well as membrane module was maintained at 37 °C with gas flow rate of 3.4 L/min. In general, both butanol flux and total flux increased with declining membrane thickness, indicating that the permeation of butanol and water molecule got enhanced. However, by calculating the water flux, it could be found that the water flux increased faster than that of butanol. Additionally, butanol separation factor was not affected markedly by the decrease of membrane thickness. Considering the compromise between the flux and separation factor of butanol (in aspect of SI), the homogeneous PDMS membrane with thickness of 100 µm was employed to evaluate the performance of VSVP process in the following studies.

**Table 1:** Separation performance of VSVP process using PDMS membranes with different thicknesses for separating butanol/water solution

Membrane thickness (µm)	Flux (g/m^2^ h)		Butanol separation factor	Butanol titer in permeate (g/L)
	Total	Butanol		
225	45.0 ± 2.7	16.6 ± 1.3	36.4 ± 2.7	339.9 ± 10.7
130	76.7 ± 3.5	26.3 ± 2.0	34.1 ± 3.8	317.2 ± 16.1
100	85.5 ± 1.6	29.4 ± 1.5	34.3 ± 2.5	318.4 ± 10.4

**Fig. 2:**
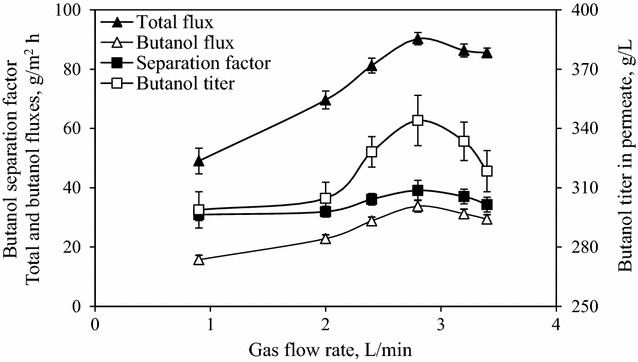
Effect of gas flow rate on butanol separation performance of VSVP process using PDMS membrane

Since butanol in feed solution is vaporized as feed for membrane permeation in VSVP process, the effect of gas flow rate on butanol separation performance was investigated during VSVP process with feed solution and membrane surroundings maintained at 37 °C. As shown in Fig. 2, with the gas flow rate varying from 0.9 to 3.4 L/min, the total flux firstly increased and subsequently remained constant, while the butanol flux declined after reaching the maximum. Maximal total and butanol fluxes of 90.3 and 33.8 g/m^2^ h, respectively, were achieved when the gas flow rate was 2.8 L/min. Moreover, the separation factor of butanol as well as recovered titer tended to reduce at gas flow rate of more than 2.8 L/min, because the increase in water diffusivity within membrane was greater than that of butanol. With increasing gas flow rate from 0.9 to 2.8 L/min, the enhanced contact time between gas bubble and feed solution contributed to more butanol and water vaporization. Simultaneously, the vaporized butanol and water have more time to contact with membrane surface for diffusion and permeation [21]. The vaporized quantities of butanol and water increased, providing greater driving force through the membrane. Therefore, the total flux and butanol flux gradually increased with increasing gas flow rate from 0.9 to 2.8 L/min. For higher gas flow rate (more than 2.8 L/min), it may take time for butanol and water molecules to adsorb and dissolve into the PDMS membrane. But some of vaporized butanol and water molecules in the membrane module have no enough time to adsorb upon the membrane surface and permeate through the membrane, especially for butanol molecule with larger diameter than water. So they were dragged back to feed vessel by higher flow rate of circulatory vapor mixture, thus resulting in the decrease of vapor stripping efficiency. Hence, slight reductions in flux and titer of recovered butanol were observed at gas flow rate of more than 2.8 L/min in Fig. 2. The results indicated that an optimal gas flow rate could enhance the vapor stripping process to achieve the higher VSVP performance and lower energy consumption. Thereby, the following VSVP experiments below were carried out at a gas flow rate of 2.8 L/min.

### Effects of feed solution and membrane surroundings temperatures on VSVP performance

For the most of pervaporation studies for butanol or ABE recovery, the feed solution and membrane surroundings are usually conducted at the same temperature due to high thermal conductivity of liquid solution [22, 23]. The 70 °C is almost the highest temperature for heating the feed solution according to literatures [24–27], and higher temperature will be close to the azeotropic point of ABE mixture. However, since the stripping vapor is used as feed, different temperatures of feed solution and membrane surroundings can be used for the VSVP process due to low thermal conductivity of gas, which may improve the performance of the VSVP process. Besides, the VSVP process may shut down because water molecules tend to be frozen upon the membrane surface when the temperature is lower than 0 °C. Therefore, the temperatures of 0–70 °C were included for investigating effects of feed solution and membrane surroundings temperatures on VSVP performance.

**Fig. 3:**
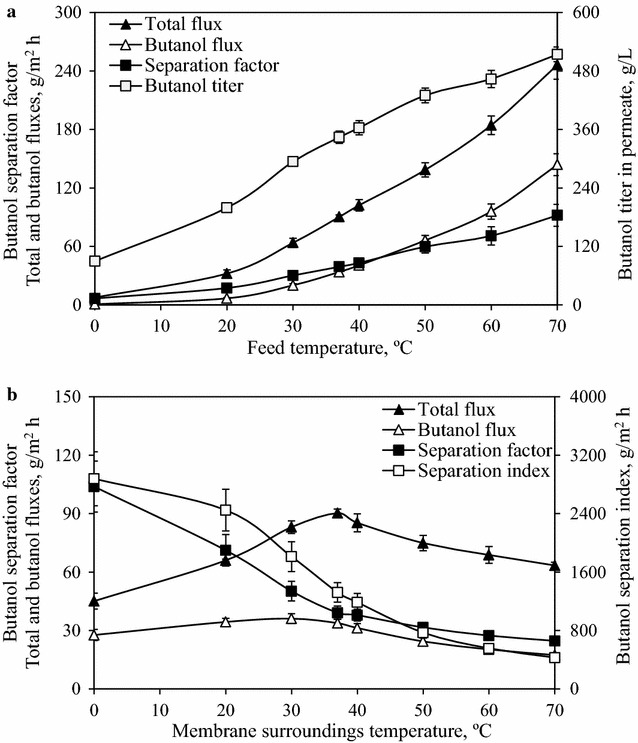
Effects of temperatures of feed solution (**a**) and membrane surroundings (**b**) on the VSVP performance using PDMS membrane

Figure 3a shows the effect of feed solution temperature on VSVP performance, in which the membrane surroundings temperature was maintained at 37 °C. With the temperature of butanol/water solution increasing from 0 to 70 °C, both total flux and butanol flux dramatically rose from 7.6 and 0.7 g/m^2^ h to 246.3 and 143.9 g/m^2^ h, respectively. Butanol titer in permeate and separation factor also increased from 89.7 g/L and 6.6  to 514.1 g/L and 91.9, respectively. It clearly indicated that the VSVP performance could be enhanced at a higher feed solution temperature. The vapor stripping effectiveness of butanol improved more with increasing feed solution temperature in comparison with that of water, which led to a significant improvement in butanol quantity in vapor mixture [28]. Moreover, at a higher temperature, the butanol and water molecules possessing higher apparent activation energy could easily and rapidly permeate through the PDMS membrane. As a result, the remarkable rises in butanol flux and separation factor were observed.

Fig. 3b shows the effect of membrane surroundings temperature on VSVP performance, in which the feed temperature was constant at 37 °C. With increasing membrane surroundings temperature, the total flux gradually increased to 90.3 g/m^2^ h at 37 °C and then deceased to 63.4 g/m^2^ h at 70 °C. Moreover, the butanol separation factor remarkably slumped from 103.9 to 24.7 as the membrane surroundings temperature increased from 0 to 70 °C. The butanol separation index declined from 2878 to 430 g/m^2^ h with increasing temperature, indicating the decrease in the effectiveness of butanol recovery. These phenomena could be attributed a lot for the changes of free volume of dense PDMS membrane, butanol, and water quantities in vapor, and vapor molecule aggregation.

**Fig. 4:**
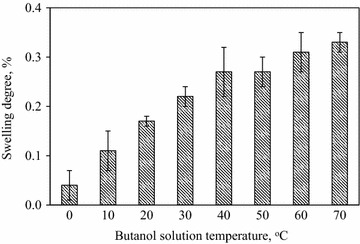
Change in the swelling degree (%) of the PDMS membrane under different temperatures

When the membrane surroundings temperature increased from 0 to 37 °C, the increase in flux depended on the increased PDMS molecular spaces and thermal motion of PDMS polymer chains, facilitating butanol and water molecules permeation. The rise in flux demonstrated above could be also elucidated by the improvement in swelling degree of the membrane with increasing temperature, as shown in Fig. 4. The homogenous PDMS membrane used in swelling experiments differ in membrane surrounding temperatures. The higher membrane surroundings temperature was maintained the more butanol and water molecules were adsorbed into the membrane, which led to the increase in swelling degree. Dobrak et al. reported similar result in ethanol recovery from ethanol/water solution using silicalite-filled PDMS membrane. They found that an increase in temperature could promote membrane swelling and consequently contributed to the increase in flux through membrane [29].

When the temperature of membrane surroundings was more than 37 °C, the temperature of membrane surroundings was higher than that of feed vapor mixture as shown in Fig. 1b (*T*_1_ < *T*_2_). The partial pressures of butanol and water in the vapor phase increased with increasing membrane surroundings temperature. In turn, the increased vapor pressure differences of butanol and water between feed solution and vapor mixture resulted in more molecules back into feed solution in order to achieve pressure equilibrium. This trend was more pronounced at a higher membrane temperature, resulting in the reduction of stripping effectiveness. Guan et al. also found this interesting phenomenon when separating dilute aqueous isopropanol solution with a composite PDMS membrane [21]. Therefore, the poorer stripping process at a higher membrane surroundings temperature decreased butanol and water quantities in vapor phase, which mainly contributed to the remarkable reduction in flux.

### Temperature-difference control for VSVP process

In order to obtain optimal VSVP performance for butanol recovery from butanol/water solution, further experiments under extreme temperatures conditions were conducted and the results are shown in Table 2. In comparison with feed solution and membrane surroundings simultaneously at 37 °C, the VSVP performance could be remarkably improved in the presence of great temperature difference. When feed solution and membrane surroundings maintained at 70 and 0 °C, respectively, the butanol flux and separation factor reached 80.7 g/m^2^ h and 142.7, respectively, and the butanol titer in permeate achieved the maximum of 591.2 g/L from ~ 15 g/L butanol in feed solution. When butanol was concentrated at such a high level, a subsequent dehydration process could be easily achieved with hydrophilic membranes, molecule sieve etc. Moreover, as shown in Table 2, the low feed solution (0 °C) and high membrane surroundings temperature (70 °C) resulted in very poor butanol flux of 0.5 g/m^2^ h and separation factor of 5.7. The demonstrating results indicated that the high temperature of membrane surroundings was not beneficial for vapor molecule permeation through the membrane. Simultaneously, the low temperature of feed solution seriously led to less vapor stripping from dilute aqueous solution, finally weakening vapor molecule permeation through the membrane.

**Table 2:** Separation performance of VSVP process for butanol recovery from binary aqueous solution under extreme temperature conditions

Temperature (°C)		Flux (g/m^2^ h)		Butanol separation factor	Butanol titer in permeate (g/L)
Feed solution	Membrane surroundings	Total	Butanol		
37	37	90.3 ± 2.1	33.8 ± 2.0	39.1 ± 3.4	344.0 ± 12.7
70	0	117.7 ± 6.1	80.7 ± 6.2	142.7 ± 15.5	591.2 ± 17.9
0	70	6.4 ± 0.5	0.5 ± 0.1	5.7 ± 0.4	78.7 ± 4.3
70	70	315.8 ± 17.5	111.7 ± 8.1	35.8 ± 2.6	326.7 ± 14.6

Since the freezing point of butanol (− 88.9 °C) was lower than that of water (0 °C), the vaporized water molecule aggregated more easily than butanol molecule at/near the membrane surface with lower temperature during VSVP process [30]. As illustrated in Fig. 1a (*T*_1_ > *T*_2_), when feed solution temperature was higher than membrane surroundings temperature, for these aggregated water molecules, the tendency to be liquefied via hydrogen bond became more obvious with increasing the temperature difference between feed solution and membrane surroundings, making it more difficult to permeate the membrane than vapor molecules. Moreover, due to the higher affinity of PDMS membrane to butanol molecules, the butanol molecules were more easily adsorbed upon the PDMS membrane surface, which then formed an adsorbed layer with less water in the initial stage of the permeation. The butanol in vapor may adsorb into the hypothetical adsorption layer and dissolve inside the PDMS membrane, along with the renewal of the adsorption layer, which made this layer-associated selective permeability. Therefore, in the VSVP process with greater temperature-difference control, the fold increase of butanol separation factor (see Table 2) could be attributed to water vapor aggregation and thickness of butanol/water adsorption layer. The great difference between vapor temperature (70 °C) and membrane surroundings temperature (0 °C) were more beneficial for butanol permeation rather than water.

### Integration of VSVP process with ABE fermentation

ABE fermentations without/with VSVP process were conducted by *Clostridium acetobutylicum* ATCC 55025 using P_2_ medium containing 93.0 g/L glucose, and the results are shown in Table 3. In general, 11.4 g/L butanol, 5.0 g/L acetone, and 1.3 g/L ethanol (17.7 g/L ABE) were produced within ~ 40 h, along with 26.8 g/L residual glucose in fermentation broth without VSVP process. The final titers of acetic and butyric acids were 1.2 and 1.7 g/L, respectively. On the contrary, for integrating the VSVP process with ABE fermentation, the VSVP process initiated at 20 h of ABE fermentation when butanol titer in fermentation broth was above 7 g/L. Glucose was completely utilized at 59 h and as high as 31.2 g/L ABE (19.6 g/L butanol, 9.8 g/L acetone and 1.9 g/L ethanol) was produced. The final titers of acetic and butyric were 1.5 and 1.7 g/L, respectively. The butanol and ABE yields improved from 0.17 and 0.27 g/g to 0.21 and 0.34 g/g, respectively. In comparison with ABE fermentation without the VSVP process, the butanol and ABE productivity of the integrated process were at the same level of 0.35 and 0.55 g/L h at the first 20 h, but significantly increased by 47.8 and 44.1% from 20 h to the end of fermentation during VSVP process, respectively. The enhanced ABE fermentation integrated with VSVP process could be accounted for in situ removal of butanol from the active fermentation broth, resulting in the mitigation of butanol inhibition to cells.

**Table 3:** Kinetics of ABE fermentation without/with VSVP process

Fermentation parameters	Batch fermentation without VSVP	Batch fermentation with VSVP
Fermentation time (h)	40	59
Glucose consumed (g/L)	66.2 ± 0.7	93.0 ± 0.7
Glucose consumption rate (g/L h)	1.66	1.57
Maximum OD_600_	3.2 ± 0.1	3.7 ± 0.1
Butanol production (g/L)	11.4 ± 0.5	19.6 ± 0.8
Acetone production (g/L)	5.0 ± 0.2	9.8 ± 0.3
Ethanol production (g/L)	1.3 ± 0.1	1.9 ± 0.1
Total ABE production (g/L)	17.7 ± 0.8	31.2 ± 1.2
Butanol productivity^a^ (g/L h)	0.35 ± 0.02 and 0.23 ± 0.02	0.35 ± 0.02 and 0.34 ± 0.02
ABE productivity^a^ (g/L h)	0.55 ± 0.02 and 0.34 ± 0.03	0.56 ± 0.02 and 0.49 ± 0.02
Butanol yield (g/g)	0.17 ± 0.01	0.21 ± 0.01
ABE yield (g/g)	0.27 ± 0.01	0.34 ± 0.02
Acetic acid produced (g/L)	1.2 ± 0.1	1.5 ± 0.1
Butyric acid produced (g/L)	1.7 ± 0.1	1.7 ± 0.1

^a^The ratio of products concentration to fermentation time, before and after VSVP process start (at 20 h of ABE fermentation)

**Fig. 5:**
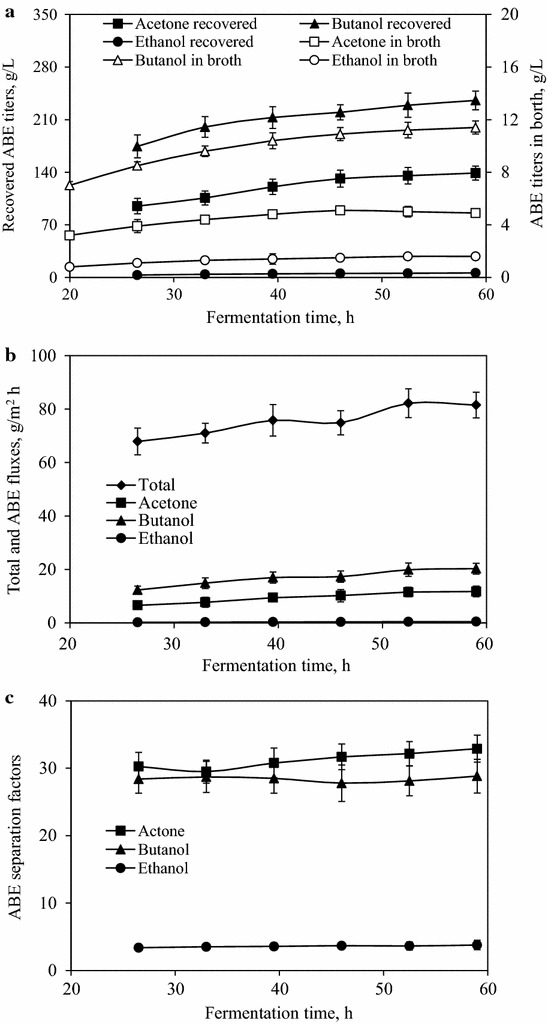
Products obtained from the VSVP process integrated with batch ABE fermentation. **a** ABE titers in broth and permeate. **b** Total and ABE fluxes. **c** ABE separation factors

The integrated VSVP process lasted for 39 h, and the performance of the VSVP process is summarized in Fig. 5. As shown in Fig. 5a, the increased ABE titer in fermentation broth generally resulted in the increase of ABE titer in permeate. After the initiation of VSVP process, the titers of butanol, acetone, and ethanol in permeate maintained in a range of 174.4–235.5 g/L, 94.9–138.9 g/L, and 3.2–6.0 g/L, respectively. In addition, the average titers of butanol, acetone, and ethanol were 212.7, 121.8, and 4.8 g/L. The butanol titer in permeate was high enough for phase separation to capture the energy-saving potential of VSVP process.

The fluxes and separation factors of ABE for ABE fermentation integrated with VSVP process were depicted in Fig. 5b, c. The fluxes of total, butanol, acetone, and ethanol were in the range of 75.1 ± 7.2, 16.3 ± 4.0, 9.2 ± 2.6, and 0.4 ± 0.1 g/m^2^ h, respectively. The separation factors of butanol, acetone, and ethanol were stable in the limited range of 27.8–29.0, 29.3–32.9, and 3.4–3.8, respectively. The greater separation factor of acetone could be contributed to its lager saturated vapor pressure at 37 °C rather than other components. In addition, the mass flux and separation factor of this VSVP process can be significantly enhanced by fabrication of the composited or mixed membrane with the PDMS polymer using super hydrophobic materials. Therefore, the ABE fermentation integrated with VSVP process is an effective way for biobutanol production.

### Comparisons of performance and energy requirement with literatures

The present study developed an alternative way to recover butanol, and PV and VSVP performances of PDMS membranes without/with supporting materials are summarized in Table 4 for comparison. The homogeneous PDMS membrane showed separation factor of 19.3–49 and undesirable butanol flux of 26.5–62.2 g/m^2^ h for separating butanol/water solution during PV process at a higher feed temperature under different conditions of membrane fabrication and system operation [31, 32], which was in agreement with previous report [33]. To improve the flux by decreasing the thickness of active PDMS layer, various supporting layers were employed, such as PE/Brass [22], polyacrylonitrile (PAN) [34] and polyvinylidene fluoride (PVDF) [23, 31]. In conventional pervaporation, the butanol separation factor of pristine PDMS membrane is usually 7.5–50 for separating model solution under different conditions in various publications [31, 32, 35–37].

**Table 4:** Comparison of pervaporation and VSVP process for butanol recovery from butanol/water solution

Method**/**membrane used	Butanol titer (g/L)		Flux (g/m^2^ h)		Butanol separation factor	Temperature (feed solution, membrane), (°C)	References
	Feed	Permeate	Total	Butanol			
PV/PDMS	15.0	216.5	287.3	62.2	19.3	80, 80	[31]
PV/PDMS	10	307.9	80	26.5	49	78, 78	[32]
PV/PDMS^a^	19.9	361.7	132	52.1	32	37, 37	[22]
PV/PDMS^a^	34.7	365.9	~ 2260	~ 904	22	50, 50	[34]
PV/PDMS^a^	9.98	~ 293.4	159.6	48.4	43.1	30, 30	[23]
PV/PDMS^a^	15.0	325.5	769.6	249	35.2	80, 80	[31]
VSVP^b^/PMP	9.98	340.6	42	12.5	58.1	35, 35	[28]
VSVP^b^/PTMSP	9.98	262.8	120	33.6	38.5	35, 35	[28]
VSVP/PDMS	15.0	344.0	90.3	33.8	39.1	37, 37	This study
VSVP/PDMS	15.0	591.2	117.7	80.7	142.7	70, 0	This study

^a^Membrane with supporting materials

^b^Non-circulatory process

The poly-4-methyl-2-pentyne (PMP) and poly-1-trimethylsilyl-1-propyne (PTMSP) membranes were investigated to recover butanol from butanol solution via non-circulatory vapor phase permeation which nitrogen as gas-carrier was supplied to generate vapor and retention substances were not circulated back to feed vessel [28]. There was no temperature-difference control in this kind of VSVP process (non-circulatory gas) with butanol separation factor of 58.1 and butanol flux of 12.5 g/m^2^ h using PMP membrane. In present work, without temperature-difference control, the close-circulating VSVP process could produce 344 g/L butanol in condensate, with butanol flux of 33.8 g/m^2^ h and separation factor of 39.1, respectively. It should be noted that the super higher butanol separation factor of 142.7 and butanol flux of 80.7 g/m^2^ h were achieved with 591.2 g/L butanol in condensate in close-circulating VSVP process with temperature-difference control using the pure PDMS membrane (see Table 4). The butanol separation factor of this VSVP process here was calculated from stripping separation factor ($\beta_{\text{strip}}$) multiplied by membrane separation factor ($\beta_{\text{memb}}$), which was obtained in single-stage process for separating butanol/water solution. The demonstrating results above showed highest recovered titer and separation factor of butanol reported to date. If using nano-particles or materials such as carbon nanotube and zeolite mixed with PDMS membrane, the separation performance could be further improved [16, 38]. In addition, the recovered butanol titer could be significantly enhanced by regulation of butanol titer in feed (see Additional file 1: Fig. S2). To be highlighted, if a small quantity of butanol and water vapor do not permeate through the membrane in the close-circulating process, they can be circulated back to feed vessel. Therefore, there were no butanol and water loss during the VSVP process, which could contribute to the enhanced product recovery efficiency and economic feasibility. In Yakovlev’s study, the nitrogen as carrier gas was used for ex situ butanol recovery, which was not suitable for the integrated ABE fermentation in consideration of gas cost and micro-environment for microbial growth [28]. In present study, the off-gas (CO_2_ and H_2_) in ABE fermentation was used for the in situ removal of butanol, which could reduce gas cost and provide a suitable micro-environment for cell growth and fermentation.

The popular ABE fermentations integrated with PV, GS or their hybrid methods for in situ removal of butanol are summarized in Table 5. The low butanol titer in permeate of ~ 65 g/L with an average separation factor of ~ 15 was obtained in continuous [39] or fed-batch [40] ABE fermentation using the PV process with PDMS membrane. When using one-stage gas stripping for butanol recovery during fed-batch ABE fermentation, the ABE titer and butanol separation factor were still very low, usually less than 200 g/L and 22, respectively, due to the lower ABE titer in broth and undesirable selectivity of butanol with gas stripping [4, 13, 17, 41, 42]. To further concentrate butanol from the aqueous phase in the condensate obtained from the first-stage gas stripping with ABE fermentation, the second-stage gas stripping was conducted, and the butanol titer in final product mixture was 420.3 g/L [4]. In addition, the second-stage pervaporation was also applied after the fed-batch ABE fermentation integrated with first-stage gas stripping [41]. In our previous study, a higher butanol titer in final products of 521.3 g/L was obtained using the PDMS membrane mixed with carbon nanotubes (CNTs). Cai et al. [17] also applied this two-stage process to achieve similar separation performance, using the PDMS/PVDF composite membrane for the second-stage PV. Because of two individual processes connection in series, one more condensation and reheating between GS and PV process would require unwanted energy consumption. Interestingly, this single-stage VSVP process using the homogeneous pure PDMS membrane exhibited the highest ever reported butanol titer of 212.7 g/L (339.3 g/L ABE) in condensate from fermentation broth containing as low as 7.0–11.4 g/L butanol in the integrated ABE fermentation system (see Table 5). The continuous butanol recovery from fermentation broth mainly contributed to the alleviation of butanol inhibition and glucose utilization consumption, thereby yielding higher butanol (ABE) productivity and yield. In comparison with gas stripping, the vaporized solvents in the VSVP process permeate through a membrane before condensation, the process of which could remarkably enhance separation factor of butanol during vapor selective permeation through the membrane. Besides, the VSVP process has various outstanding advantages such as easiness for operation, no removal of nutrients from medium, and no harmfulness to cells. Furthermore, only vaporized solvents that contact with both sides of the membrane in VSVP process usually are beneficial for generating high concentrated solvents, which overcomes the drawbacks of membrane fouling and undesirable separation performance induced by unfavorable substances contact with membrane surface in PV [14, 43, 44].

**Table 5:** Comparison of separation performance and energy required for in situ ABE recovery

Recovery method	Butanol in broth (g/L)	Solvent in condensate (g/L)		Butanol separation factor	A/E/W composition (g/g-butanol)^c^	Temperature, (broth/feed, condensation), (°C)	Energy required (kJ/g-butanol)^e^	References
		Butanol	ABE					
PV	2.7–10.1	35–64	63–117	13.7–15.7	0.69/0.13/16.6	35, − 2	85.0	[39]
PV	3.2–6.8	~ 60.7	~ 74.1	7–19	0.19/0.02/15.0	36, 0^d^	76.4	[40]
GS	6.0–12.4	115–160	140–195	15.8–22.2	0.23/0.04/6.20	37, 1	32.6	[42]
GS	8–13	150.5	195.9	~ 17.4	0.26/0.04/5.12	37, 2	27.3	[13]
GS–GS^a^	3.5–14.6	175.6	227.0		0.24/0.05/4.18	37, 2	22.6	[4]
		420.3	593.2	–	0.15/0.03/0.60	37, 2	3.9	
GS–PV^a^	7.7–14.2	155.6	199.9		0.23/0.05/4.92	37^d^, 2	26.2	[41]
		521.3	622.9	97.8	0.13/0.01/0.32	80, − 196	5.2	
GS–PV^b^	10–12	108.3	177.6		0.44/0.19/7.37	37, − 5	39.0	[17]
		482.6	706.7	76.8–92.3	0.33/0.10/0.41	~25, − 196	5.1	
VSVP	7.0–11.4	212.7	339.3	27.8–29.0	0.56/0.02/2.88	37, − 196	19.6	This study

^a^The second-stage recovery was employed to separate solvent in aqueous phase after GS process

^b^No phase separation between two-stage separation

^c^For each gram of butanol recovered in single-stage separation process, the corresponding quantities of acetone (A), ethanol (E), and water (W) were contained in condensate. And the individual amounts were calculated from ABE titer in condensate

^d^The data in parentheses are not mentioned in literatures, and speculated according to operation conditions

^e^The data of energy required for in situ ABE recovery are not provided in the cited literatures, and these values were calculated according to the same rule as described in the text. Additionally, only vaporization energy of 19.34 kJ/g-butanol was calculated by Cai et al. [17], and the energy requirement should be 39.0 kJ/g-butanol if in consideration of both vaporization and condensation (other 0.32 kJ/g-butanol for cooling liquid)

For better understanding energy consumption for PV, GS, hybrid strategies, and alternative VSVP process, the energy requirements for in situ butanol recovery during ABE fermentation are also compared in Table 5. The energy or heat required mainly included the evaporation heat (latent heat) and condensation heat (latent heat for liquefying and sensible heat for cooling liquid), which is on the basis of the state changes of ABE and water molecules separated. The energy requirement for evaporating components separated in PV or GS process, *E*^evap^, is calculated as mentioned in many literatures [14, 17, 21]. Moreover, during condensation, the energy must be removed to condense the permeate vapor, which is approximately equal to the energy required for evaporation (*E*^cond^= − *E*^evap^). And the sensible heat for cooling liquid does not involve phase change. It should be pointed out that our calculation of energy consumption is based on the ABE titer in permeate and system temperature (for fermentation/feed and condensation), for the individual integration of ABE fermentation with recovery processes. And when separating each gram of butanol in condensate, the corresponding quantities of acetone, ethanol, and water were also separated at the same time (see “A/E/W composition” column in Table 5).

As illustrated in Table 5, the PV processes with PDMS membrane required 76.4–85.0 kJ/g-butanol from active fermentation broth [39, 40], which was calculated on the grounds of the same consideration condition with other processes. The energy requirement for PV was also summarized with a broad range of 2–145 kJ/g in our previous publication [13], but the calculation was not clearly specified in the cited references. The energy requirement for in situ butanol recovery by single GS or first-stage GS processes was estimated at 22.6–39.0 kJ/g, due to the variation of process conditions and butanol concentrations in fermentation broth [4, 13, 17, 41, 42]. For the second-stage recovery by GS or PV, the energy requirement was in the range of 3.9–5.2 kJ/g [4, 17, 41]. The energy required is extremely governed by the water content in product recovered, since water possesses higher enthalpy of vaporization and specific heat capacity than that of ABE. Therefore, a recovery process offering high separation factor of ABE can significantly reduce the energy consumption. Remarkably, the VSVP process required 19.6 kJ/g of energy consumption, generating a highly concentrated product with single-stage recovery. It should be noted that the conventional distillation process requires ~ 36 kJ/g for butanol recovery from the dilute butanol solution (~ 1%, w/v), which was nearly equal to the energy content of butanol (36 kJ/g). Thus, it is clear that the VSVP process can provide an energy-efficient way for butanol recovery integrated with ABE fermentation, which is superior to other processes above. Other techniques including extraction and adsorption were also evaluated for their potential of reduction in energy consumption [13, 45], but it’s difficult for them to obtain a high recovered butanol titer through single-stage recovery process. In summary, the close-circulating VSVP process with temperature-difference control demonstrated an advanced technique for biobutanol recovery with low energy requirement, especially for integration with ABE fermentation.

## Conclusions

A novel close-circulating vapor stripping-vapor permeation (VSVP) process was developed to recover biobutanol with temperature-difference control. The separation performance of the VSVP process was remarkably affected by the individual temperature of feed solution and membrane surroundings, thereby yielding the butanol flux and separation factor of 80.7 g/m^2^ h and 142.7 with the optimal temperature-difference control. Furthermore, this VSVP process integrated with ABE fermentation generated 212.7 g/L butanol (339.3 g/l ABE) in condensate, with the reduced energy consumption of 19.6 kJ/g-butanol, which was much superior to other recovery techniques. This advanced VSVP process can effectively avoid membrane fouling and facilitate biobutanol production when integrating with ABE fermentation.

## Methods

### Strain and media

*Clostridium acetobutylicum* ATCC 55025 was used for ABE fermentation. The Clostridial growth medium (CGM) for seed culture and P_2_ medium for ABE fermentation were described in previous study [4, 13], and incubated at 37 °C for ~ 16 h with no agitation until active growth was observed. To eliminate the dissolved oxygen, all solutions were purged with nitrogen for 0.5 h through a sterile 0.2 µm filter and sterilized at 0.2 MPa (absolute pressure) and 121 °C for 15 min.

### Fabrication of homogeneous PDMS membrane

The base solution from the Sylgard^®^ 184 silicone elastomer kit (Dow Corning, USA) was mixed with the curing agent in a ratio of 10:1 (w/w) by employing pentane as the solvent to dilute the mixture. The membranes were fabricated according to our previous publication and then assembled into a membrane module [31].

### Swelling degree of PDMS membrane

Since the membrane swelling may impact butanol permeation, the swelling study was conducted by immersing PDMS membranes in butanol–water solutions under given condition for 24 h to determine their wet weights $(W_{1} )$ (g). There were two test variables: butanol concentration (0–70 g/L) and solution temperature (0–70 °C). After drying the membranes in oven for 24 h, the dry membranes were also weighted $\left( {W_{0} } \right)$ (g). The swelling degree (SD) value of PDMS membranes was calculated by following equation [46, 47]:

1 $${\text{SD}} = \frac{{W_{1} - W_{0} }}{{W_{0} }} \times 100\%$$

### Vapor stripping-vapor permeation with PDMS membrane

The butanol/ABE solution and ABE fermentation broth were used to evaluate the VSVP separation performance under different conditions. The butanol/water model solution contained ~ 15 g/L butanol. As illustrated in Fig. 1, the liter-sized feed vessel and membrane module were placed in different thermostatic water baths. The air in sealed system was circulated between feed vessel and membrane module to generate feed vapor mixture using a peristaltic pump. To reduce vapor condensation in tubes, the pipeline between feed vessel and membrane module was wrapped by thermal insulation jacket. Vacuum was < 100 Pa provided on the permeation side of PDMS membrane via a vacuum pump. The recovered permeate was collected in a cold trap. After the system was stabilized, samples were withdrawn at internals of 2 h and then weighted. In addition, all experiments were performed in triplicate.

### Batch fermentation and integrated VSVP process start-up

For ABE fermentation without/with VSVP, the whole system was sparged with nitrogen for 0.5 h through a sterile 0.2 µm filter to maintain an anaerobic environment. Then, the bioreactor containing 0.9 L P_2_ medium was inoculated with 100 mL of actively growing cell and controlled at agitation rate of 150 rpm and 37 °C. The pH was maintained at 5 by automatic addition of 2 N NH_3_·H_2_O. When butanol titer in fermentation broth was about 7.0 g/L, the VSVP process was initiated to continuously recover ABE solvents with a circulation rate of 2.8 L/min for ABE fermentation integrated with VSVP system. The samples were withdrawn periodically from the bioreactor and cold trap for the analysis of fermentation kinetics and recovered products.

### Analytical methods

Cell biomass, glucose and all metabolic products in the fermentation broth were analyzed according to our previous study [48]. The separation performance of VSVP process was characterized by flux and separation factor. And total flux $(J_{T} )$ (g/m^2^ h) was calculated as follow [47]:

2 $$J_{T} = \frac{W}{At},$$

where *W* is the weight of recovered permeate (g), *A* is the membrane area (m^2^) and *t* is the time (h). The flux of component $i$ $(J_{i} )$ is equal to its mass fraction in permeate $(y_{i} )$ multiplied by total flux $(J_{T} )$:

3 $$J_{i} = J_{T} y_{i}$$

In pervaporation process, the separation factor was obtained by following equation [47]:

4 $$\beta_{i} = \frac{{y_{i} /(1 - y_{i} )}}{{x_{i} /(1 - x_{i} )}}$$

And in this hybrid process, vapor stripping followed by membrane vapor permeation, the total separation factor for component $i$ $\beta_{i}$ was composed of vapor stripping separation factor (liquid-to-vapor transition separation factor,$\left( {\beta_{\text{strip}} } \right)$ and membrane separation factor $\left( {\beta_{\text{memb}} } \right)$. Thus total separation factor could be obtained as follow [21, 28]:

5 $$\beta_{i} = \beta_{\text{strip}} \cdot \beta_{\text{memb}} = \frac{{y_{{{\text{strip}},i}} /\left( {1 - y_{{{\text{strip}},i}} } \right)}}{{x_{i} /\left( {1 - x_{i} } \right)}} \cdot \frac{{y_{i} /\left( {1 - y_{i} } \right)}}{{y_{{{\text{strip}},i}} /\left( {1 - y_{{{\text{strip}},i}} } \right)}} = \frac{{y_{i} /\left( {1 - y_{i} } \right)}}{{x_{i} /\left( {1 - x_{i} } \right)}},$$

where $x_{i}$ and $y_{{{\text{strip}},i}}$ are the mass fractions of component $i$ in feed solution and vapor mixture, respectively.

In the VSVP process with PDMS membrane, the separation index for component $i$ ${\text{SI}}_{i}$ [g/m^2^ h] depended on the joint action of both partial flux and separation factor, which could be calculated by the equation [34, 47, 49–51]

6 $${\text{SI}}_{i} = J_{i} \beta_{i}$$

## Additional file

**Additional file 1: Fig. S1.** Change in the swelling degree (%) of the PDMS membrane under different butanol titers in feed. **Fig. S2.** Effect of feed butanol titer on the VSVP performance using PDMS membrane.

## Abbreviations

GS: gas stripping
PV: pervaporation
VSVP: vapor stripping-vapor permeation
ABE: acetone butanol ethanol
CGM: clostridial growth medium
PDMS: polydimethylsiloxane
PVDF: polyvinylidene fluoride
PAN: polyacrylonitrile
PMP: poly-4-methyl-2-pentyne
PTMSP: poly-1-trimethylsilyl-1-propyne
CNTs: carbon nanotubes
