# Supplementary material for: A novel close-circulating vapor stripping-vapor permeation technique for boosting biobutanol production and recovery
Source: Biotechnol Biofuels. 2018 May 4;11:128. doi: 10.1186/s13068-018-1129-5 (PMC5934881; doi:10.1186/s13068-018-1129-5)
Supplement: Supplementary file 1 — Additional file 1: Fig. S1. Change in the swelling degree (%) of the PDMS membrane under different butanol titers in feed. Fig. S2. Effect of feed butanol titer on the VSVP performance using PDMS membrane. [file 13068_2018_1129_MOESM1_ESM.docx]

**﻿ELECTRONIC SUPPLEMENTARY MATERIAL**

**For “a novel close-circulating vapor stripping-vapor permeation technique for boosting biobutanol recovery”**

Fig. S1. Change in the swelling degree (%) of the PDMS membrane under different butanol titers in feed.

Fig. S2. Effect of feed butanol titer on the VSVP performance using PDMS membrane.
